# circ_0046599 Promotes HCC Progression by Competing with miR-1322 to Enhance NT5DC2 Expression

**DOI:** 10.7150/jca.103661

**Published:** 2025-03-31

**Authors:** Xiaobo Zhang, Suxin Li, Luhao Li, Dingyang Li, Huashan Zhao, Xiaole Gao, Xiaowei Dang

**Affiliations:** 1 Department of Hepatobiliary and Pancreatic Surgery, the First Affiliated Hospital of Zhengzhou University, Zhengzhou, China.; 2 Key Laboratory of Precision Diagnosis and Treatment in General Surgical (Hepatobiliary and Pancreatic) Diseases of Health Commission of Henan Province, Zhengzhou, China.; 3 Henan Province Engineering Research Center of Minimally Invasive Diagnosis and Treatment of Hepatobiliary and Pancreatic Diseases, Zhengzhou, China.; 4 Budd-Chiari Syndrome Diagnosis and Treatment Center of Henan Province, Zhengzhou, China.; 5 Department of Hepatobiliary and Pancreatic Surgery, College of Clinical Medicine of Henan University of Science and Technology, the First Affiliated Hospital of Henan University of Science and Technology, Luoyang, China.; 6 Department of Pathology, the First Affiliated Hospital of Zhengzhou University, Zhengzhou, China.; 7 Department of Pharmacy, the First Affiliated Hospital of Zhengzhou University, Zhengzhou, China.

**Keywords:** circ_0046599, miR-1322, NT5DC2, HCC

## Abstract

**Background**: This study aimed to investigate the impact of circ_0046599 on hepatocellular carcinoma (HCC).

**Methods:** Analysis of the GEO dataset identified circ_0046599 as significantly upregulated in HCC, and its impact on cell proliferation, apoptosis, migration, and invasion was assessed. Bioinformatics and dual-luciferase assays identified miR-1322 as a target of circ_0046599, which in turn regulates NT5DC2 expression. In vitro and in vivo experiments confirmed the ceRNA mechanism of circ_0046599 in HCC.

**Results:** circ_0046599 was significantly upregulated in HCC, and predicts a worse survival in HCC patients. Increased expression of circ_0046599 promoted HCC cell proliferation, migration, invasion, and inhibited apoptosis. circ_0046599 bound to miR-1322, which exerted a tumor-suppressive effect in HCC cells. miR-1322 targeted NT5DC2, and circ_0046599 regulated the expression of NT5DC2 by competitively binding to miR-1322. Modulation of NT5DC2 expression affected the oncogenic role of circ_0046599. In *in vivo* experiments, inhibition of circ_0046599 suppressed the growth of xenograft tumors by upregulating miR-1322 expression and suppressing NT5DC2.

**Conclusion**: circ_0046599 promoted the progression of HCC by competitively binding to miR-1322 and regulating the expression of NT5DC2.

## Introduction

Hepatocellular carcinoma (HCC) is a prevalent form of cancer with a bleak outlook, making it a significant global health concern^1^, ^2^. Typically, HCC patients face challenges in early diagnosis due to the absence of specific symptoms, which contributes to an unfavorable prognosis^3^. The available treatment options for HCC encompass resection, local ablative therapies, and liver transplantation. However, these therapies are not suitable for patients in advanced stages^4^ who often exhibit resistance to treatment and a low response rate^5^. To address these circumstances, effective biomarkers are required for HCC to aid in early detection, prognosis assessment, and prediction of treatment response.

Roughly 97% of the human genome is transcribed into non-coding (nc) RNAs, which fulfill vital roles in tumorigenesis despite lacking protein-coding abilities^6^. Among these regulatory ncRNAs, circular RNAs (circRNAs) have gained increasing prominence as critical modulators of gene expression and cellular functions^7^. CircRNAs are single-stranded RNA molecules that are covalently closed and highly stable. They are generated from pre-mRNAs through a process called back-splicing and frequently participate in tumorigenesis^8^. This unique group of transcripts can also sequester microRNAs (miRNAs), inhibiting their suppressive effects on other transcripts. This phenomenon is known as competitive endogenous RNAs (ceRNAs)^9^, ^10^. CircRNAs have been identified as important biomarkers or regulators in Liver hepatocellular carcinoma (LIHC, also called HCC) and are associated with various processes, including cellular development, metastasis, and drug resistance^11^, ^12^. miR-1322 has been demonstrated to function as a tumor suppressor gene in various cancers through the ceRNA mechanism^13^, ^14^. For instance, under the regulation of circKDM1B, miR-1322 inhibits the proliferation, migration, and invasion of HCC cells and promotes apoptosis by suppressing PRC1^15^. The 5'-Nucleotidase Domain Containing 2 (NT5DC2) protein, belonging to the NT5DC family, features a haloacid dehalogenase motif at its N-terminus. NT5DC2 has been linked to both attention-deficit/hyperactivity disorder and bipolar disorder^16^. Recent findings indicate that NT5DC2 interacts with tyrosine hydroxylase (TH), influencing TH catalytic activity and thereby modulating catecholamine synthesis^17^. However, the specific role of NT5DC2 in HCC development and progression remains largely unexplored.

According to the analysis of the GSE97332 dataset in this study, a circRNA known as hsa_circ_0046599 was identified as significantly upregulated in HCC tissues. Notably, elevated levels of circ_0046599 have been observed in tumor tissues from patients with HCC, indicating a correlation with poor outcomes^18^. However, the precise role of circ_0046599 in HCC development and the specific molecules involved require further investigation. Subsequent bioinformatic analyses suggested that circ_0046599 may act as a sponge for miR-1322, thereby restoring the expression of NT5DC2. It is worth noting that miR-1281 has been associated with anti-proliferative and anti-metastatic effects on cancer cells^19^. Based on these findings, it is speculated that there may exist a circ_0046599/miR-1322/NT5DC2 axis involved in the progression of HCC.

## Material and methods

### Bioinformatics analysis

The circRNA gene expression microarray GSE97332 for HCC was downloaded from the GEO database (https://www.ncbi.nlm.nih.gov/geo/query/acc.cgi?acc=GSE97332), which included cancer tissues and corresponding adjacent tissues from seven HCC patients. The downstream miRNAs of circ_0046599 were predicted using online miRNA prediction tools circinteractome (https://circinteractome.nia.nih.gov/) and circbank (http://www.circbank.cn/). Based on the Starbase miRNA prediction tool, the downstream target genes of candidate miRNAs were predicted, and the top 100 upregulated genes were selected using the UALCAN database. The intersection of these two sets of genes was visualized using a Venn diagram.

### Cell culture and transient transfection

Human HCC cell lines Hep-3B, SNU-387, and normal Transformed Human Liver Epithelial-2 (THLE-2) were obtained from FuHeng BioLogy (Shanghai, China). All cells were cultured in Roswell Park Memorial Institute 1640 medium (Gibco, Grand Island, NY, USA) supplemented with 1% double antibody (sigma-Aldrich, St. Louis., MO, USA) under standard conditions of 5% CO_2_ and 37°C.

Overexpression of NT5DC2 (NT5DC2) and circ_0046599 siRNA (si-circ_0046599#1, si-circ_0046599#2, si-circ_0046599#3) along with their respective controls (vector, si-NC) were designed and constructed by Ribobio (Guangzhou, China). In addition, miR-1322 mimic and inhibitor were purchased from Jima Corporation (Suzhou, China). All cell transfections were performed using Lipofectamine 2000 reagent (Invitrogen, Carlsbad, CA, USA) according to the manufacturer's protocol.

### FISH assay

Fluorescence in situ hybridization (FISH) was conducted on two HCC cell lines. Antisense and sense probes targeting the circ_0046599 sequence were synthesized (RiboBio, Guangzhou, China). Hybridization was performed using a fluorescence in situ hybridization kit (Beyotime, Jiangsu, China). HCC cells were seeded onto glass chamber slides, and 24 hours post-transfection, cells were fixed with 4% paraformaldehyde (Thermo Scientific, Rockford, IL, USA) for 10 minutes, followed by three washes with PBS (Sigma-Aldrich, USA) for 5 minutes each. Permeabilization was carried out at room temperature using 0.5% Triton X-100/PBS for 20 minutes, followed by three additional PBS washes for 5 minutes each. Subsequently, cells were incubated with the specific probes at 37°C overnight. The nuclei were stained with DAPI, and the staining results were observed using a confocal fluorescence microscope.

### Clinical samples

Fresh hepatocellular carcinoma (HCC) samples, along with their para-carcinoma tissues, were collected from the First Affiliated Hospital of Zhengzhou University. The diagnosis of HCC was confirmed by two seasoned pathologists, and the specimens were immediately preserved in liquid nitrogen to maintain RNA integrity. Additionally, to evaluate the diagnostic potential of circ_0046599, plasma samples were obtained from HCC patients (n=18) and healthy individuals (n=27). Baseline patient information was retrieved from the hospital information management system, and survival follow-ups were conducted, with mortality as the primary endpoint.

### qRT-PCR

Total RNA was isolated from tumor tissues or cells using TRIzol reagent (Invitrogen) following the manufacturer's protocol. The purity and concentration of RNA samples were checked using NanoDrop ND-1000 (Thermo Fisher Scientific, Waltham, MA, USA). Reverse transcription of RNA into cDNA was performed using SuperScript II reverse transcriptase (Invitrogen). qRT-PCR was carried out on an ABI 7500 qPCR system using the AceQ qPCR SYBR Green Master Mix (Vazyme, Nanjing, China) reagent. β-actin was used as the reference gene for circRNA and mRNA, and U6 was used as an internal control for miRNA expression levels. Gene expression was quantified using the 2^-ΔΔCt^ method. Specific primer sequences were arranged in Table 1.

### circRNA stability detection

RNase R (Beyotime, Beijing, China) was used to treat total RNA. Briefly, equal aliquots of RNA extracted from Hep-3B and SNU-387 cells were divided into two parts: one part was subjected to RNase R digestion, and the other part was treated with RNase R reaction buffer as a control. For RNase R digestion, 2 μg of total RNA was mixed with 2 μl of 10× RNase R reaction buffer and 2 μl of RNase R (20 U/μl). For the Control group, DEPC-treated water was used instead of RNase R. The RNA samples were incubated at 37°C for 30 minutes, followed by qRT-PCR to detect the expression of circ_0046599 and B3GNTL1 mRNA.

### Cell viability assay

The CCK-8 assay kit (Beyotime) was used to assess cell proliferation. HCC cell lines were seeded in 96-well plates at a density of 3000 cells per well. After culturing for 0, 24, 48, and 72 hours, 10 μl of CCK-8 solution was added to each well, followed by incubation for an additional 2 hours. The absorbance at 450 nm was measured using a microplate reader (Bio-Tek Company, Winooski, VT, USA).

### Colony formation assay

HCC cells were seeded in 6-well plates at a density of 500 cells per well and incubated in a standard culture condition for 14 days. The cells were fixed with methanol (Solarbio, Beijing, China) and stained with 0.1% crystal violet (Beyotime) for 1 hour. Visible colonies were manually counted under a microscope (Olympus, Tokyo, Japan).

### TUNEL staining assay

Tunel Cell Apoptosis Detection Kit (Servicebio, Wuhan, China) was used to analyze apoptotic cells as per specifications. Briefly, the transfected cells were immobilized utilizing 4% paraformaldehyde for 20 min, stained with TUNEL reagent for 1 h at 37°C, and finally incubated with DAPI under light protection. The images were observed using a fluorescence microscope (Olympus), and cells were computed using Image J software.

### Scratch wound healing assay

Transfected cells were seeded in 6-well plates and grown to 80% to 90% confluence. A single-cell layer was scratched using the tip of a 10 µl pipette, and PBS was used to wash away cell debris. The medium without FBS was added, and cells were further cultured for 24 hours. Images were obtained at 0 and 24 hours at the same scratch position, and the wound width was calculated using ImageJ software.

### Transwell invasion assay

Transwell assays with pre-coated Matrigel in 24-well chambers (8 µm pore size, Corning, NY, USA) were performed to assess cell invasion. In brief, a cell suspension containing 5×10^4^ transfected cells in 300 μl of serum-free medium was seeded in the upper chamber. Simultaneously, 700 μl of growth medium containing 10% FBS was placed in the lower chamber. After incubation at 37°C for specified time, the cells that invaded to the lower chamber were fixed with 4% paraformaldehyde and stained with 0.5% crystal violet. Subsequently, the invaded cells were photographed and counted in five randomly selected fields under an inverted light microscope (Olympus).

### Western blot

Total protein was extracted from cells using RIPA lysis buffer (Beyotime) supplemented with protease and phosphatase inhibitor cocktails (Roche). The membranes were incubated overnight at 4°C with primary antibodies, including NT5DC2 (1:1000, bs-19491R, bioss, Beijing, China) and GAPDH (1:5000, ab181602, Abcam) as a loading control. After washing with TBST, the membranes were incubated with horseradish peroxidase (HRP)-conjugated secondary antibodies (1:5000, Cell Signaling Technology) for 2 hours at room temperature. Protein bands were visualized using an enhanced chemiluminescence (ECL) detection solution (BeyoECL Star, Beyotime). The relative expression of proteins was quantified using ImageJ software (NIH, Bethesda, MD, USA), with GAPDH serving as the internal loading control to normalize protein expression levels.

### Dual-luciferase reporter assay

The potential binding sites of circ_0046599 with miR-1322 obtained from circBank and the potential binding sites of NT5DC2 with miR-1322 obtained from Starbase were identified, and respective mutant sequences were designed. The 3' UTR sequences containing circ_0046599 WT/ circ_0046599 MT or NT5DC2 WT/NT5DC2 MT were inserted into the pGL3 luciferase reporter gene vector (Promega, Madison, WI, USA) to generate corresponding luciferase reporter plasmids. These reporter plasmids were transfected with miR-1322 mimic/miR-NC into Hep-3B and SNU-387 cells. After 48 hours, luciferase activity was determined using the Dual-Luciferase Reporter Assay System (Promega).

### *In vivo* tumorigenicity assay

All animal experiments were approved by our institution's Animal Ethics Committee and conducted in accordance with our institution's guidelines for the care and use of laboratory animals. The lentiviral short hairpin RNA (sh-circ_0046599) and contrast sh-NC were constructed by Ribobio. Male nude mice aged 4-6 weeks were obtained from Beijing Vital River Laboratory Animal Technology Co., Ltd. (Beijing, China) and randomly divided into two groups: sh-NC and sh-circ_0046599, with 5 mice in each group. Tumor xenografts were established by subcutaneous injection of 200 μl (1×10^6^ cells) of transfected Hep-3B cells mixed with 30% Matrigel. Tumor size was measured every 7 days, and tumor volume was calculated using the formula: (length × width^2^ × 0.5). After 4 weeks, mice were euthanized with an overdose of pentobarbital sodium (150 mg/kg), and the harvested tumor tissues were used for subsequent experiments.

### Immunohistochemistry (IHC)

Antigen retrieval was performed by heating the sections in citrate buffer (pH 6.0) at 95°C for 20 minutes. After cooling, the sections were washed in phosphate-buffered saline (PBS) and blocked with 3% hydrogen peroxide for 10 minutes to quench endogenous peroxidase activity. The tissue sections were incubated overnight at 4°C with primary antibodies against Ki67 (1:200, GB111499, Servicebio) and NT5DC2 (1:200, bioss). The next day, sections were washed with PBS and incubated with a biotinylated secondary antibody for 30 minutes at room temperature, followed by incubation with streptavidin-HRP (horseradish peroxidase) for another 30 minutes. The staining was visualized using diaminobenzidine (DAB) as the chromogen, and counterstaining was performed with hematoxylin (Sigma) for nuclear staining. Sections were examined and photographed using an Axiophot optical microscope (Zeiss, Oberkochen, Germany). The percentage of positive staining was determined using Image J.

### Statistical analysis

Statistical analysis was performed using GraphPad Prism 8.02 software (GraphPad, San Diego, CA, USA). Differences between two groups were evaluated using paired or unpaired *t*-tests. One-way ANOVA or two-way ANOVA was used for comparisons among multiple groups, followed by Tukey's post hoc test. All data from at least 3 duplicates were exhibited as mean ± standard deviation (SD). *P*-value < 0.05 was considered statistically significant.

## Results

### hsa_circ_0046599 is significantly upregulated in HCC

**Figure 1:**
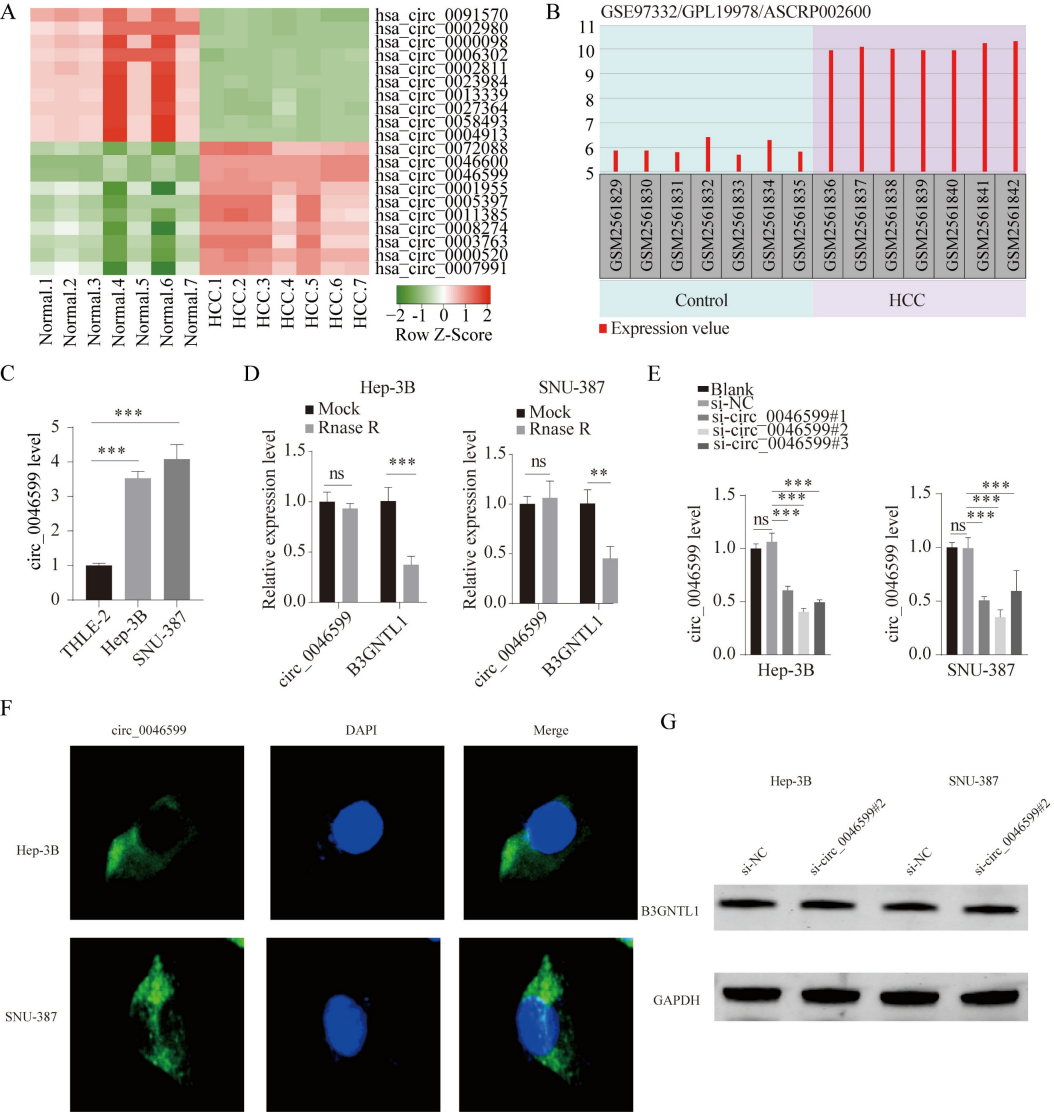
** hsa_circ_0046599 exhibits significantly high expression in HCC.** A. Heatmap of the ten most upregulated circRNAs and ten most downregulated circRNAs in circRNA gene expression microarray GSE97332. Red represented upregulated circRNAs and green represented downregulated circRNAs. B. The expression of circ_0046599 in HCC tissues (n=7) and normal controls (n=7) was shown according to GSE97332 dataset. C. qRT-PCR analysis of circ_0046599 expression levels in THLE-2, Hep-3B, and SNU-387 cell lines. D. qRT-PCR analysis of circ_0046599 and linear B3GNTL1 expression levels in Hep-3B and SNU-387 cells after RNase R treatment. E. Construction of three siRNAs targeting circ_0046599 for transfection into Hep-3B and SNU-387 cells, followed by qRT-PCR analysis of circ_0046599 expression levels. F. Detection of the distribution of circ_0046599 by fluorescence in situ hybridization (FISH). G. Effect of circ_0046599 on the protein expression of its host gene. **P<0.01, ***P<0.001, ns: no significance.

First, we downloaded the circRNA gene expression microarray GSE97332 for HCC from the GEO database, which included cancer tissues and corresponding adjacent tissues from seven HCC patients. As depicted in **Fig. 1A**, the top 10 up-regulated circRNAs and 10 down-regulated circRNAs in GSE97332 was analyzed by heat map. Differential expression analysis revealed that hsa_circ_0046599 was significantly upregulated in HCC tissues compared to adjacent tissues (**Fig. 1A**). According to GSE97332 database, the level of circ_0046599 was overexpressed in HCC tumor tissues in contrast to that in adjacent non-tumor tissues (**Fig. 1B**). To further validate the expression level of circ_0046599 in HCC, we analyzed its expression in the liver stellate cell line THLE-2 and HCC cell lines Hep-3B and SNU-387. We found that circ_0046599 was significantly increased in HCC cell lines (**Fig. 1C**). To confirm the circular characteristic of circ_0046599, RNase R, a 3'-5' exoribonuclease, was used. RNase R can degrade linear RNA but not circular RNA structures. The results showed that circ_0046599 was resistant to RNase R digestion, while the linear B3GNTL1 was degraded (**Fig. 1D**). Therefore, to further validate the relationship between circ_0046599 and the malignant biological behavior of HCC cells, we designed three siRNAs targeting circ_0046599 and found that si-circ_0046599#2 had the highest knockdown efficiency (**Fig. 1E**). We further investigated the subcellular localization of circ_0001583 using FISH methods with a probe specific to circ_0001583. The results demonstrated that circ_0001583 was present in the cytoplasm of both hepatocellular carcinoma cell lines (**Fig. 1F**). Additionally, we examined the impact of circ_0046599 on its host gene. Following the knockdown of circ_0046599, no significant effect was observed on the protein expression of B3GNTL1 (**Fig. 1G**).

### Knockdown of circ_0046599 inhibits the malignant biological behavior of HCC cells

**Figure 2:**
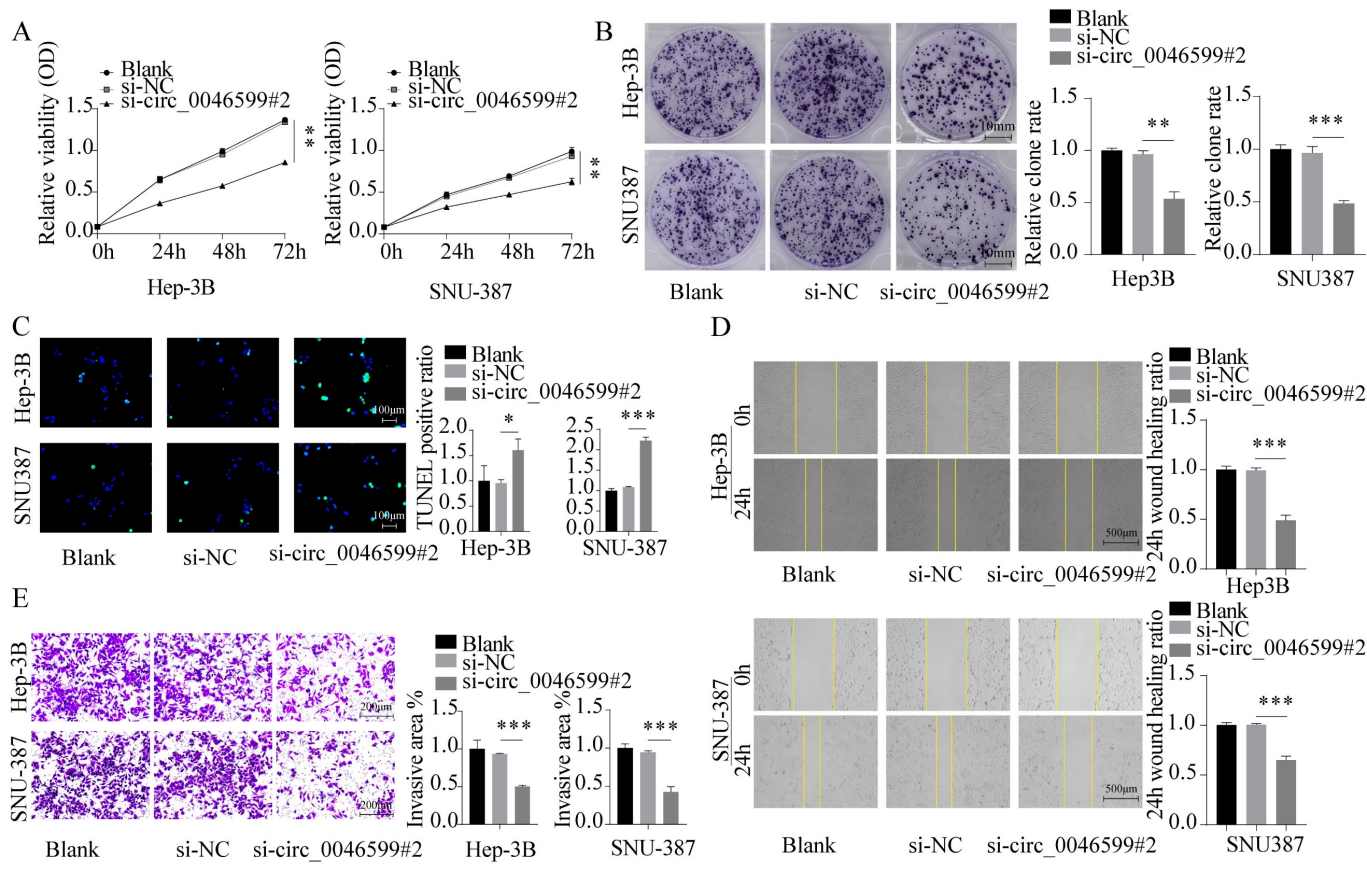
** Knockdown of circ_0046599 suppresses the malignant biological behavior of HCC cells.** A. CCK-8 assay to measure the cell viability of Hep-3B and SNU-387 cells. B. Colony formation assay to assess the proliferative capacity of Hep-3B and SNU-387 cells. C. TUNEL assay to determine the apoptotic ratio in Hep-3B and SNU-387 cells. D. Scratch assay to evaluate the 24-hour healing rate of Hep-3B and SNU-387 cells. E. Transwell invasion assay to examine the invasive ability of Hep-3B and SNU-387 cells. *P<0.05, **P<0.01, ***P<0.001.

We first assessed the cell viability and proliferation capacity of Hep-3B and SNU-387 cells using CCK-8 and colony formation assays, respectively. We found that the growth and proliferation abilities of HCC cell lines were significantly weakened in cells with circ_0046599 knockdown (**Fig. 2A-B**). Furthermore, TUNEL staining was performed to detect the number of apoptotic cells in Hep-3B and SNU-387 cells, and it was found that the number of apoptotic cells increased significantly under si-circ_0046599 treatment (**Fig. 2C**). Moreover, scratch and Transwell assays were conducted to evaluate the migration and invasion abilities of Hep-3B and SNU-387 cells. The results showed that the migration rate of HCC cells with low circ_0046599 expression was significantly reduced at 24 hours (**Fig. 2D**), and the number of cells invading the lower chamber of the Transwell system was also significantly reduced (**Fig. 2E**). These results indicate that circ_0046599 is closely associated with the malignant biological behavior of HCC cells.

### Circ_0046599 targets miR-1322

**Figure 3:**
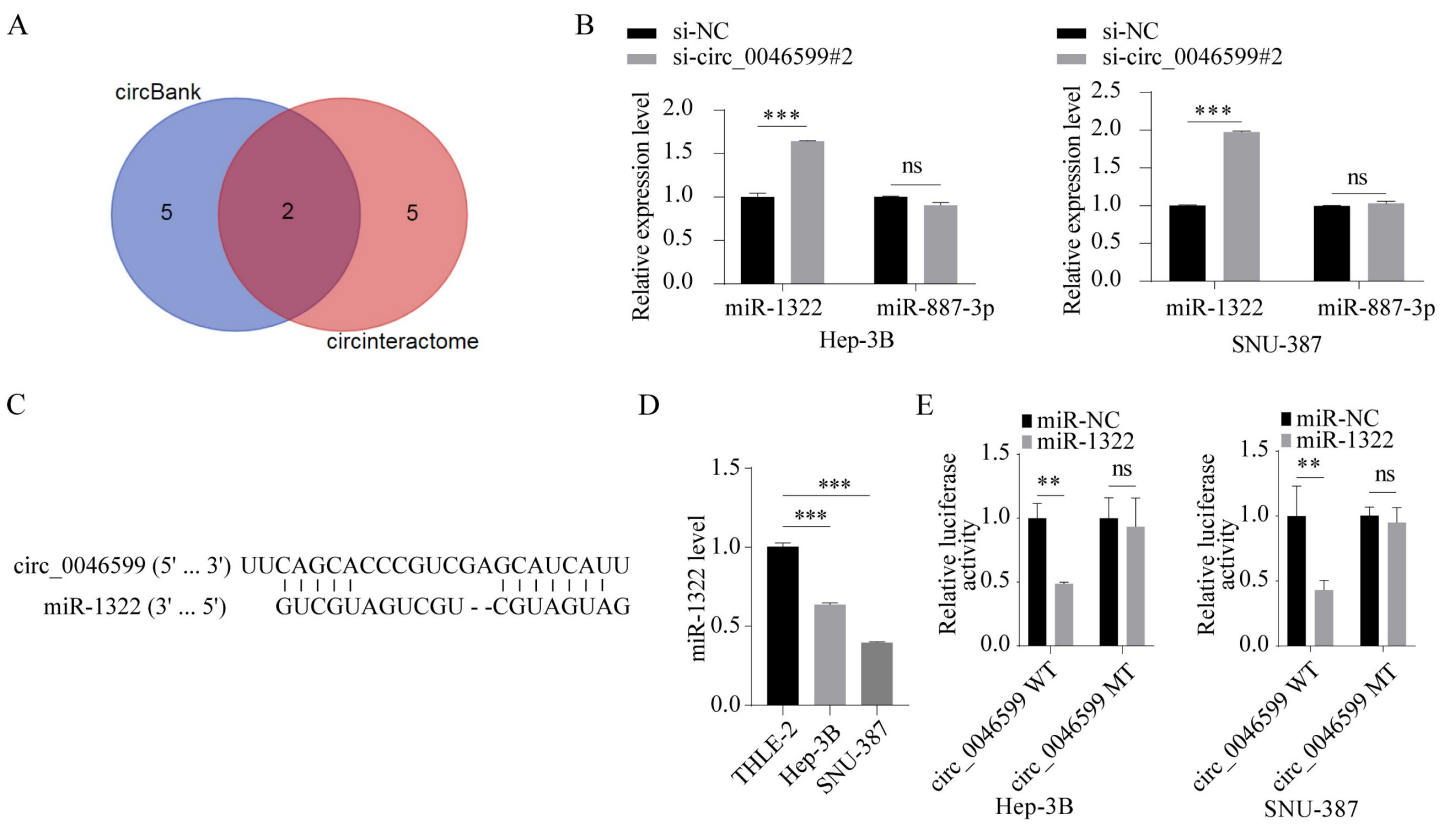
** Circ_0046599 targets miR-1322.** A. Prediction and cross-screening of downstream miRNAs of circ_0046599 using miRNA online prediction tools (circinteractome; circbank). B. qRT-PCR analysis of miR-1322 and miR-887-3p expression levels in Hep-3B and SNU-387 cells with circ_0046599 knockdown. C. Schematic diagram exhibited the target binding sites between circ_0046599 and miR-1322. D. qRT-PCR analysis of miR-1322 expression levels in THLE-2, Hep-3B, and SNU-387 cell lines. E. Dual-luciferase reporter assay to validate the targeting interaction between miR-1322 and circ_0046599. **P<0.01, ***P<0.001, ns: no significance.

To determine the downstream mechanism of circ_0046599, we predicted the downstream miRNAs of circ_0046599 using miRNA prediction tools (circinteractome; circbank), and identified two common miRNAs, miR-1322 and miR-887-3p (**Fig. 3A**). Subsequently, we detected the expression levels of miR-1322 and miR-887-3p in Hep-3B and SNU-387 cells with circ_0046599 knockdown, and found that the expression of miR-1322 was significantly increased in cells with circ_0046599 knockdown, while the expression level of miR-887-3p remained unchanged (**Fig. 3B**). The putative complementary region between circ_0046599 and miR-1322 was exhibited in **Fig. 3C**. Moreover, the expression level of miR-1322 in HCC cells was significantly lower than that in the THLE-2 cells (**Fig. 3D**). To further confirm the binding relationship between circ_0046599 and miR-1322, luciferase reporter vectors containing the binding sites of circ_0046599 and miR-1322 were constructed. Co-transfection of miR-1322 mimic and the wild-type reporter vector resulted in the most significant reduction in luciferase activity (**Fig. 3E**). These results indicate that circ_0046599 can target and bind to miR-1322.

### Restoration of HCC cell activity by miR-1322 inhibitor

**Figure 4:**
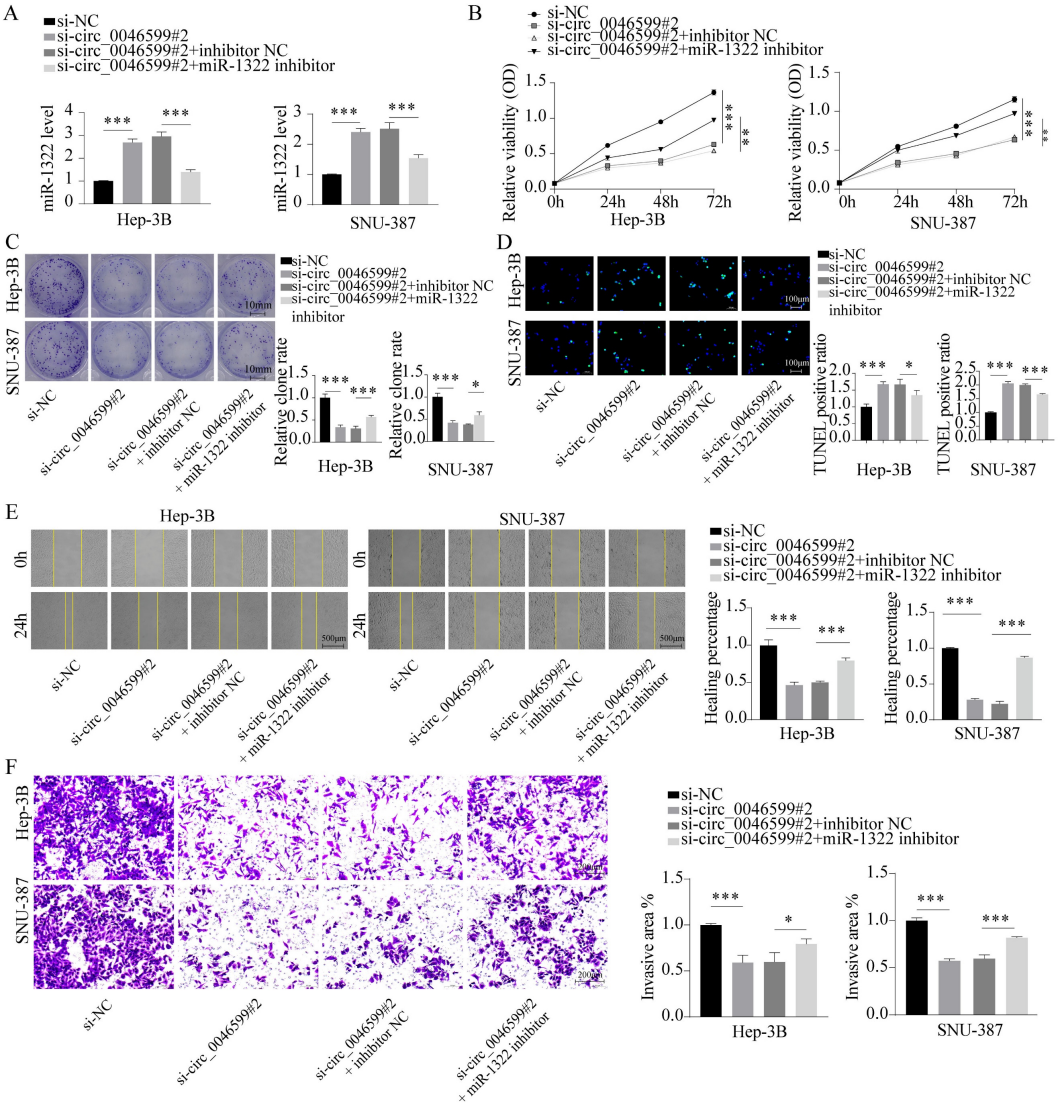
** miR-1322 inhibitor restores the activity of si-circ_0046599 HCC cells.** A. qRT-PCR analysis of miR-1322 expression levels in Hep-3B and SNU-387 cells with low circ_0046599 expression transfected with miR-1322 inhibitor. B. CCK-8 assay to measure the cell viability of Hep-3B and SNU-387 cells. C. Colony formation assay to assess the proliferative capacity of Hep-3B and SNU-387 cells. D. TUNEL assay to determine the apoptotic ratio in Hep-3B and SNU-387 cells. E. Scratch assay to evaluate the 24-hour healing rate of Hep-3B and SNU-387 cells. F. Transwell invasion assay to examine the invasive ability of Hep-3B and SNU-387 cells. *P<0.05, **P<0.01, ***P<0.001.

To further confirm whether miR-1322 can affect the malignant biological behavior of HCC cells by binding to circ_0046599, we further transfected Hep-3B and SNU-387 cells with the miR-1322 inhibitor in cells with low circ_0046599 expression (**Fig. 4A**). The results showed that inhibiting miR-1322 expression further promoted the proliferation and growth activity of Hep-3B and SNU-387 cells with low circ_0046599 expression (**Fig. 4B-C**), and the number of TUNEL-positive cells decreased significantly, indicating a decrease in apoptotic cells after inhibiting miR-1322 expression (**Fig. 4D**). Moreover, the scratch and Transwell invasion assays showed that knocking down miR-1322 in cells with low circ_0046599 expression significantly restored the migration and invasion abilities of Hep-3B and SNU-387 cells (**Fig. 4E-F**).

### miR-1322 targets and binds to NT5DC2

**Figure 5:**
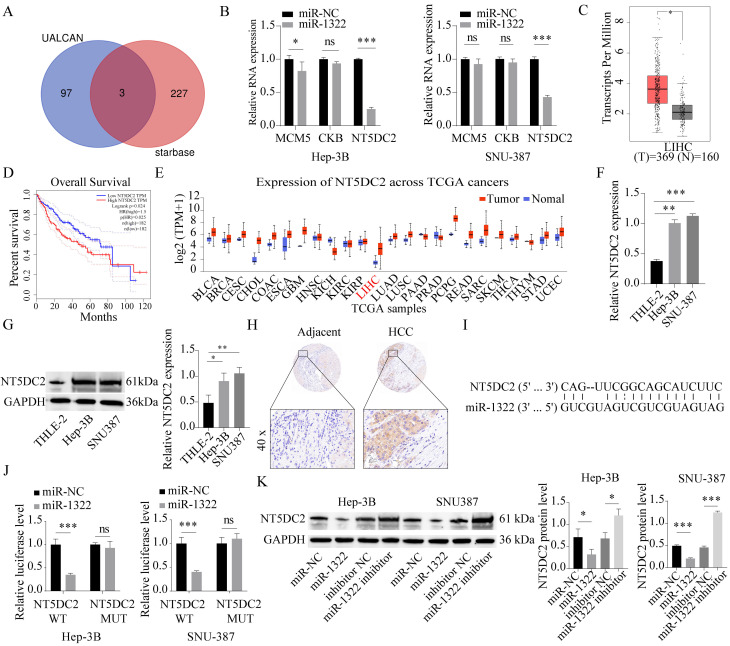
** miR-1322 targets NT5DC2.** A. Prediction of downstream target genes of candidate miRNAs using the online tool Starbase, and selection of the top 100 upregulated genes from the UALCAN database, with an intersection analysis using a Venn diagram. B. qRT-PCR analysis of MCM5, CKB, and NT5DC2 mRNA expression levels in cells overexpressing miR-1322. C. Expression levels of NT5DC2 in HCC or pan-cancer based on analysis using the GEPIA database. D. Kaplan-Meier analysis showing the correlation between NT5DC2 expression levels and survival in GEPIA-HCC patients. E. Expression levels of NT5DC2 in different TCGA cancers analyzed by UALCAN database. F-G. qRT-PCR and Western blot analysis of NT5DC2 expression levels in THLE-2, Hep-3B, and SNU-387 cell lines. H. IHC staining intensity of NT5DC2 in HCC tumor tissues and adjacent normal tissues using a tissue array. I. Schematic diagram displayed the putative binding sites between miR-1322 and NT5DC2. J. Dual-luciferase reporter assay was proceeded to confirm the interaction between miR-1322 and NT5DC2. K. Western blot analysis of NT5DC2 expression levels in Hep-3B and SNU-387 cells transfected with miR-1322 mimic or inhibitor. *P<0.05, **P<0.01, ***P<0.001, ns: no significance.

Based on miRNA target gene prediction tools (Starbase), we predicted the downstream target genes of the candidate miRNAs. In addition, the UALCAN database was used to screen the top 100 upregulated genes, and a Venn diagram was generated to identify the intersection between the two sets, resulting in three common candidate mRNAs (MCM5, CKB, NT5DC2) (**Fig. 5A**). Subsequently, we performed qRT-PCR to determine the expression of these three genes in cells overexpressing miR-1322. We found that the expression level of NT5DC2 was most significantly decreased in cells overexpressing miR-1322 (**Fig. 5B**). Furthermore, we discovered that NT5DC2 was significantly upregulated in HCC tissues through analysis of the GEPIA and UALCAN databases (**Fig. 5C and 5E**). In addition, in GEPIA database, patients with high NT5DC2 expression had a shorter overall survival period (**Fig. 5D**). We further analyzed the expression levels of NT5DC2 in the liver stellate cell line THLE-2 and HCC cell lines Hep-3B and SNU-387, and found that NT5DC2 expression was significantly increased in HCC cell lines (**Fig. 5F-G**). Tissue microarray results showed that NT5DC2 was highly expressed in HCC tissues compared to adjacent normal tissues (**Fig. 5H**). **Fig. 5I** showed the complementary binding sequence between NT5DC2 3'UTR and miR-1322. Moreover, the Dual-luciferase reporter assay results demonstrated a significant binding relationship between miR-1322 and NT5DC2 (**Fig. 5J**). To further clarify the regulatory relationship between miR-1322 and NT5DC2, we transfected miR-1322 mimic or inhibitor into Hep-3B and SNU-387 cells, and the results indicated that miR-1322 negatively regulated the expression of NT5DC2 (**Fig. 5K**).

### miR-1322 binding to NT5DC2 inhibits the malignant biological behavior of HCC cells

**Figure 6:**
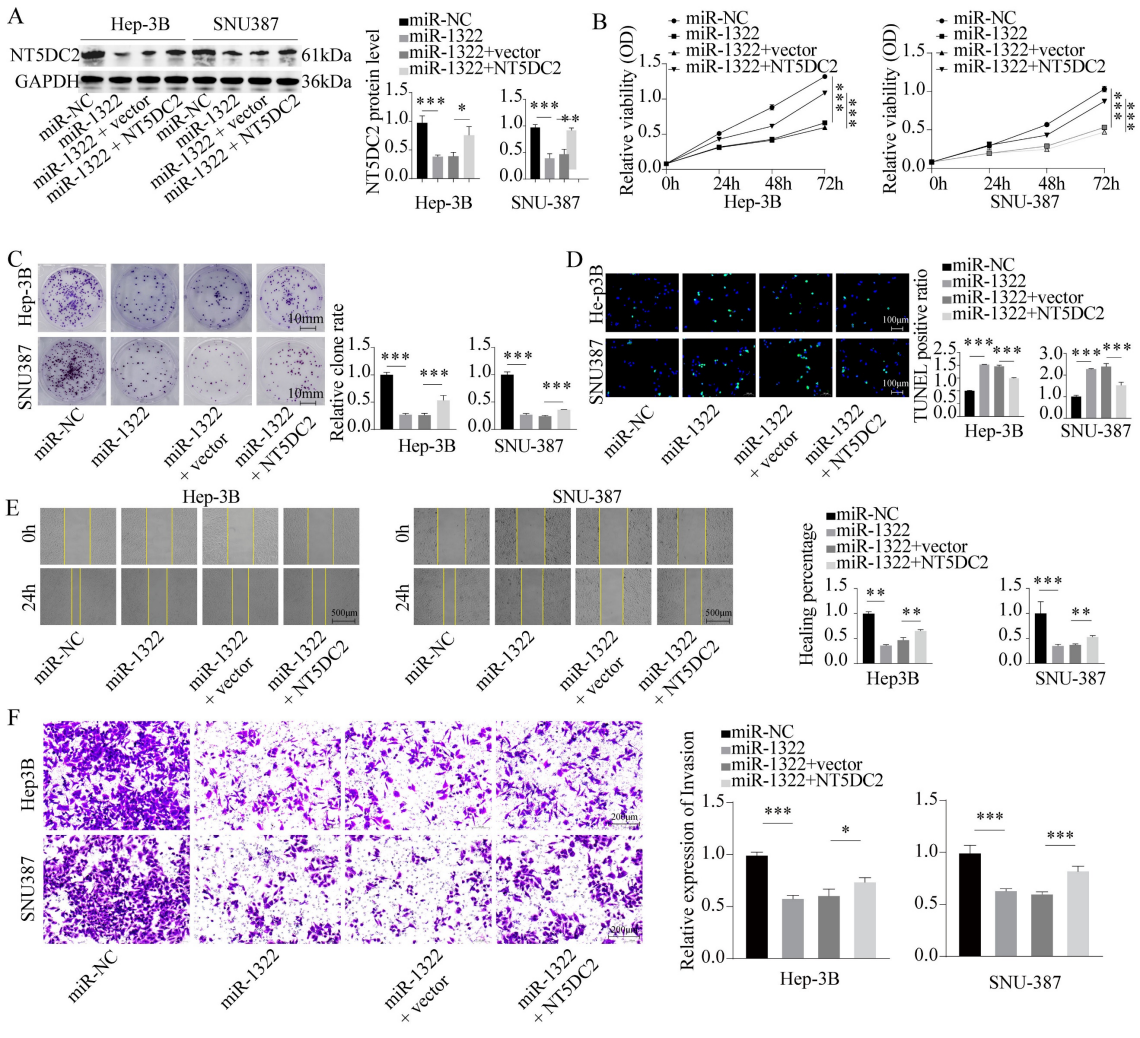
** miR-1322 binding to NT5DC2 suppresses the malignant biological behavior of HCC cells.** A. Transfection of miR-1322 mimic and overexpression of NT5DC2 in Hep-3B and SNU-387 cells, followed by Western blot analysis of NT5DC2 expression levels. B. CCK-8 assay to measure the cell viability of Hep-3B and SNU-387 cells. C. Colony formation assay to assess the proliferative capacity of Hep-3B and SNU-387 cells. D. TUNEL assay to determine the apoptotic ratio in Hep-3B and SNU-387 cells. E. Scratch assay to evaluate the 24-hour healing rate of Hep-3B and SNU-387 cells. F. Transwell invasion assay to examine the invasive ability of Hep-3B and SNU-387 cells. *P<0.05, **P<0.01, ***P<0.001.

To confirm the impact of miR-1322 and NT5DC2 on the malignant biological behavior of HCC cells, we transfected Hep-3B and SNU-387 cells with miR-1322 mimic and overexpressed NT5DC2 in the presence of miR-1322 (**Fig. 6A**). We found that the proliferation and growth activity of HCC cells were significantly attenuated after miR-1322 overexpression, while the malignant proliferation ability of cells was restored after NT5DC2 overexpression (**Fig. 6B-C**). Additionally, we observed a significant increase in the apoptotic ratio in cells overexpressing miR-1322, while the number of TUNEL-positive cells decreased significantly after NT5DC2 overexpression (**Fig. 6D**). Scratch and Transwell invasion assays yielded consistent results (**Fig. 6E-F**). These results indicate that miR-1322 acts as a tumor suppressor by inhibiting the expression of NT5DC2 and thereby suppressing the malignant biological behavior of HCC cells.

### *In vivo* effects of circ_0046599 on the malignant biological behavior of HCC cells

**Figure 7:**
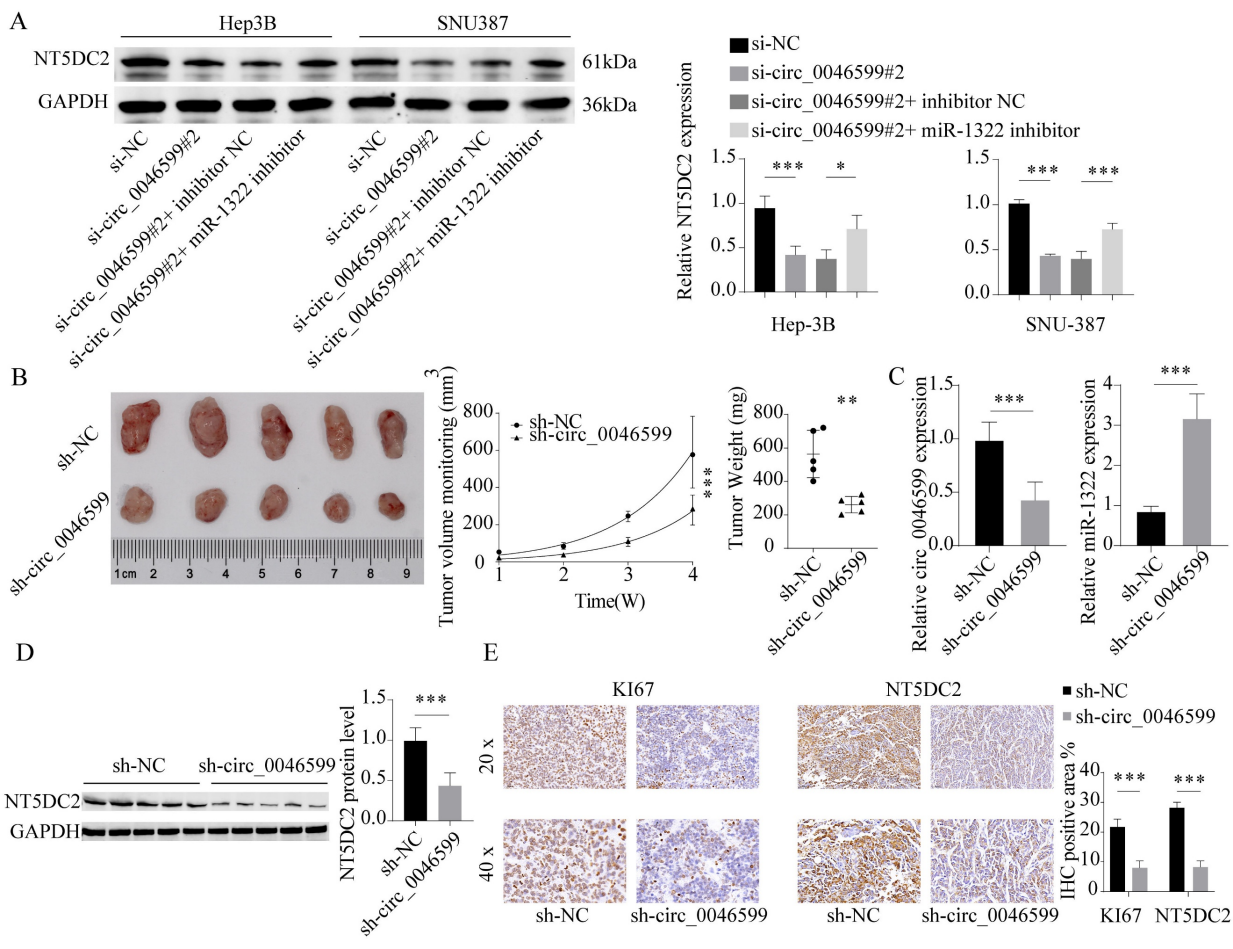
** Malignant biological behavior of circ_0046599 and HCC cells *in vivo*.** A. Western blot analysis of NT5DC2 protein expression levels in Hep-3B and SNU-387 cells after intervention with circ_0046599 and miR-1322 expression. B. Construction of stable Hep-3B cell lines with low circ_0046599 expression, followed by subcutaneous injection of cells into nude mice and measurement of tumor growth rate. C-D. qRT-PCR and Western blot analysis of circ_0046599, miR-1322, and NT5DC2 expression levels in xenograft tumors. E. IHC staining of KI67 and NT5DC2 in tumor tissues. *P<0.05, **P<0.01, ***P<0.001.

We observed a significant decrease in the protein expression level of NT5DC2 in cells with circ_0046599 knockdown, and further transfection of miR-1322 inhibitor led to a significant increase in NT5DC2 expression in these cells (**Fig. 7A**). This result suggests that circ_0046599 can regulate NT5DC2 expression through miR-1322 and influence the malignant biological behavior of HCC cells. To further validate the impact of circ_0046599 on the *in vivo* growth of HCC cells, we established stable Hep-3B cells with low circ_0046599 expression and implanted them into nude mice via subcutaneous injection. We found that cells with low circ_0046599 expression exhibited significantly reduced in vivo growth activity (**Fig. 7B**), and the expression level of miR-1322 was significantly increased in the tumor, while the expression level of NT5DC2 was significantly decreased (**Fig. 7C-D**). Finally, we performed IHC staining to examine the staining intensity of Ki67 and NT5DC2 in tumor tissues, and found that the staining intensity of Ki67 and NT5DC2 was significantly reduced in xenograft tumor tissues formed by cells with low circ_0046599 expression (**Fig. 7E**). In summary, circRNA_0046599 can competitively bind to miR-1322, thereby promoting the expression of NT5DC2 and facilitating the malignant biological behavior of HCC cells.

### The potential of circ_0046599 as a diagnostic and prognostic biomarker in HCC

**Figure 8:**
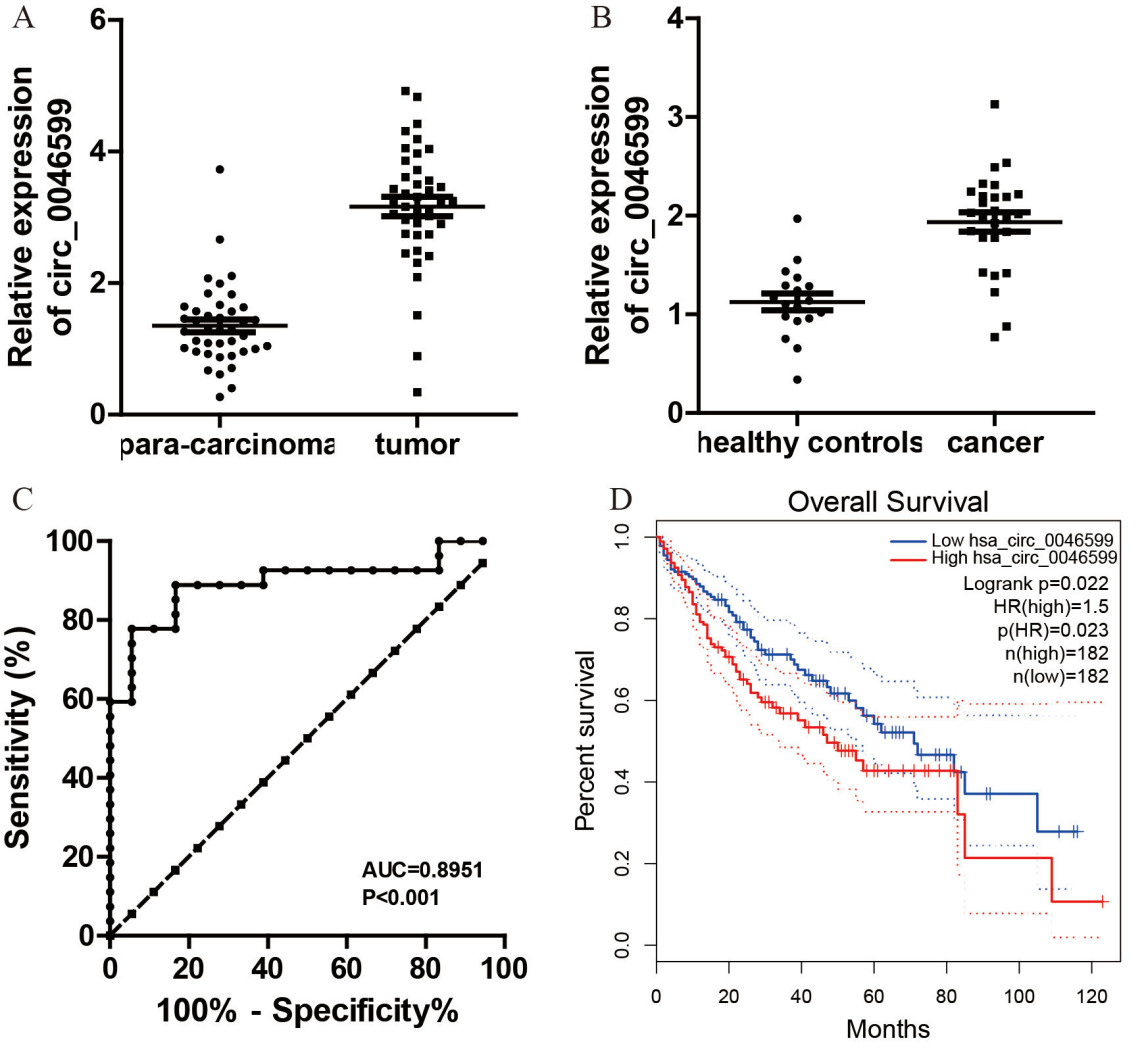
**Elevated circ_0046599 Expression in HCC and its Prognostic Implications.** A. Quantitative RT-PCR analysis of circ_0046599 in paired HCC and adjacent normal tissues. B. Plasma circ_0046599 expression in HCC patients compared to healthy controls. C. ROC Curve analysis for plasma circ_0046599 diagnostic utility in HCC. D. Kaplan-Meier survival analysis of HCC patients based on circ_0046599 expression levels in TCGA database. Statistical Significance *p < 0.05 **p < 0.01 ***p < 0.001.

Initially, 40 pairs of HCC and adjacent normal tissues were collected to assess circ_0046599 expression. qRT-PCR analysis revealed a significant upregulation of circ_0046599 in HCC tissues compared to para-carcinoma tissues (**Fig. 8A**). Additionally, plasma circ_0046599 levels were measured and found to be significantly higher in HCC patients than in healthy controls (**Fig. 8B**). The ROC curve analysis, based on plasma circ_0046599 levels, indicated an AUC of 0.8951, suggesting its efficacy as a diagnostic biomarker for HCC (**Fig. 8C**). Furthermore, high circ_0046599 expression was associated with shorter survival times in TCGA cohort (**Fig. 8D, E**).

## Discussion

HCC stands as the primary cause of cancer-related deaths in China^20^. While tobacco prevention plays a crucial role, it alone is insufficient to overcome this malignancy. Therefore, it is imperative to identify changes in key driver genes and investigate new molecular mechanisms associated with tumorigenesis. These efforts are crucial to provide additional opportunities and enhance the survival rates of patients. In this context, we present a novel circ_0046599/miR-1322/NT5DC2 axis that may be intricately connected to the pathogenesis of HCC.

CircRNAs are generated through a process called back-splicing, wherein a downstream 5' splice site is connected to an upstream 3' splice site, spanning one or multiple exons. Unlike linear RNAs, circRNAs lack free ends, which contributes to their enhanced stability^7^. These circular transcripts exhibit diverse biological functions and serve as versatile molecular regulators. Additionally, they have emerged as novel diagnostic and prognostic markers in various human cancers, including LIHC^21^. For instance, hsa_circ_0000977 promotes HCC progression by acting as a sponge for miR-141-3p, thereby upregulating the expression of SRY transcription factor^22^, ^23^. Besides, circRNA_104075 enhances HCC growth and metastasis by sponging miR-582-5p, leading to increased expression of FOXO1 and activation of the β-catenin signaling pathway^24^. Moreover, circRNA_001575 acts as a competitive endogenous RNA (ceRNA) for miR-7, thus upregulating the expression of RAF1 and promoting HCC proliferation and invasion^25^. Circular RNA hsa_circ_0000190 functions as a ceRNA for miR-646, resulting in the upregulation of PD-1 and PD-L1 expression, and promoting immune evasion in HCC^26^. In this study, we utilized the GSE97322 dataset from the GEO database to validate that circ_0046599 is the most upregulated circRNA in LIHC. As previously mentioned, circ_0046599 has been identified as a potential prognostic biomarker in LIHC^18^. In our study, we observed elevated expression of circ_0046599 in tumor tissues from HCC patients, indicating an advanced tumor stage, lymph node metastasis, poor tumor differentiation, and an unfavorable prognosis. However, the precise role of this circRNA in human cancers has received limited attention thus far. In our paper, through gain- and loss-of-function experiments, we discovered that circ_0046599 exhibits significant promotive effects on the growth and metastasis of HCC cells both *in vitro* and* in vivo*.

The subcellular localization of circ_0046599 in the cytoplasm implies its potential role as a competitive endogenous RNA (ceRNA) for other transcripts. Through bioinformatic analysis and subsequent assays, it was indicated that circ_0046599 has the ability to bind to miR-1322, which exhibits low expression in HCC samples. Notably, in gastric cancer cells, the sequestration of miR-1322 by long non-coding RNA (lncRNA) GIHCG has been shown to enhance proliferation and migration^19^. Similarly, in glioma cells, downregulation of miR-1322 by lncRNA01857 has been reported to promote malignancy^27^. In this study, we confirmed that upregulation of miR-1322 resulted in reduced proliferation and aggressiveness and apoptosis in HCC cells. Additionally, integrated bioinformatic analyses suggested that miR-1322 targets NT5DC2 in HCC. Besides, Zhang H et al explores the role of miR-1322 in prostate cancer and its regulatory effects on apoptosis (programmed cell death) and the cell cycle. It suggests that miR-1322 may have oncogenic properties and is involved in the dysregulation of key genes related to cancer progression^28^. NT5DC2 (5'-nucleotidase domain-containing 2) is a gene that encodes a protein involved in nucleotide metabolism. Zhang *et al.* identifies and characterizes the NT5DC2 gene based on an expressed sequence tag (EST) library of human saliva secretion. While it does not directly examine the role of NT5DC2 in cancer, it provides basic information about the gene and its expression patterns^29^-^31^.

The elevated levels of circ_0046599 in the plasma of HCC patients indicate its potential as a non-invasive biomarker for early detection, monitoring, and prognosis in HCC. Current diagnostic methods for HCC have limitations in sensitivity and specificity; hence, plasma circ_0046599 offers a complementary tool for diagnosis and disease surveillance. The correlation between high plasma circ_0046599 levels and poor prognosis also supports its role in risk stratification and personalized patient management, ultimately contributing to more effective therapeutic strategies and improved outcomes for HCC patients.

It is important to note that our in vivo experiments primarily focused on assessing tumor growth, and we did not include a metastasis model. This represents a limitation of the current study, as the metastatic potential of circ_0046599 remains unexplored. The absence of a metastasis model implies that our conclusions regarding the role of circ_0046599 in HCC progression are primarily related to tumor growth rather than the full metastatic cascade. Future studies should aim to include metastasis assays to fully elucidate the role of circ_0046599 in HCC metastasis and provide a more comprehensive understanding of its function in cancer progression. Despite this limitation, our findings provide important insights into the oncogenic role of circ_0046599 and its regulatory network involving miR-1322 and NT5DC2, highlighting its potential as a therapeutic target in HCC.

## Conclusion

In conclusion, our study suggests that circ_0046599 could potentially serve as a novel prognostic biomarker for HCC. Its upregulation was found to enhance the growth and metastasis of cancer cells by acting as a sponge for miR-1322 and activating NT5DC2. These findings provide new insights into the management of HCC, indicating that circ_0046599 and NT5DC2 may hold promise as therapeutic targets for HCC treatment.

## Figures and Tables

**Table 1:** The list of primer sequences for PCR.

Name		Primers for PCR (5'-3')
hsa_circ_0046599	Forward	CACCGAACGATACACACGTT
	Reverse	GCAACCAATGATGCTCGAC
Linear B3GNTL1	Forward	TGGACCAGAGTCTCCTGCTGTA
	Reverse	TTGCCAGCGTTCCAGATGGTGA
miR-1322	Forward	GTTTGGGATGATGCTGCTG
	Reverse	GTGCAGGGTCCGAGGT
miR-887-3p	Forward	TGCGCGTGAACGGGCGCCATCC
	Reverse	CCAGTGCAGGGTCCGAGGTATT
β-actin	Forward	CTTCGCGGGCGACGAT
	Reverse	CCACATAGGAATCCTTCTGACC
U6	Forward	CTCGCTTCGGCAGCACA
	Reverse	AACGCTTCACGAATTTGCGT
NT5DC2	Forward	AGCCTGGAGTTTGACCAAGCAC
	Reverse	CAGGACAGCAAACGTCTCATCC
MCM5	Forward	GACTTACTCGCCGAGGAGACAT
	Reverse	TGCTGCCTTTCCCAGACGTGTA
CKB	Forward	GGCAAGCATGAGAAGTTCTCGG
	Reverse	ACCAGCTCCACCTCTGAGAAGC

**Table 2:** Transfected gene sequences.

Gene Name		Sequence (5'→3')
si-NC	Sense	UUCUCCGAACGUGUCACGUTT
	Anti-sense	ACGUGACACGUUCGGAGAATT
si-circ_0046599#1	Sense	GCUCCUAACCCAGGAUGACGUTT
	Anti-sense	ACGUCAUCCUGGGUUAGGAGCTT
si-circ_0046599#2	Sense	GCAGCUCCUAACCCAGGAUGATT
	Anti-sense	UCAUCCUGGGUUAGGAGCUGCTT
si-circ_0046599#3	Sense	CCUAACCCAGGAUGACGUCAUTT
	Anti-sense	AUGACGUCAUCCUGGGUUAGGTT
miR-1322 mimic (miR-1322)		GAUGAUGCUGCUGAUGCUG
miR-NC		UCACAACCUCCUAGAAAGAGUAGA
miR-1322 inhibitor		CAGCAUCAGCAGCAUCAUC
anti-NC (anti-miR-NC)		CAGUACUUUUGUGUAGUACAA
Vector		pcDNA3.1
sh-circ_0046599	Sense	CCGGGCAGCTCCTAACCCAGGATGACTCGAGTCATCCTGGGTTAGGAGCTGCTTTTTG
Scrambled sh-NC	Sense	CCGGCAACAAGATGAAGAGCACCAACTCGAGTTGGTGCTCTTCATCTTGTTGTTTTTG
